# Ginkgo Biloba as a niche theme cognitive enhancer agent, 1420 dokumen of Scopus database. A bibliometric study from 1988 to 2024

**DOI:** 10.12688/f1000research.160416.3

**Published:** 2025-10-15

**Authors:** ARMAN YURISALDI SALEH, Dwi Arwandi Yogi Saputra, Riezky Valentina, Tirta Darmawan Susanto

**Affiliations:** 1 Neurology Department Faculty of Medicine, Universitas Pembangunan Nasional (UPN) Veteran Jakarta, Jakarta, Special Capital Region of Jakarta, 12450, Indonesia; 2 Department of Public Health Sciences, Faculty of Medicine, Universitas Pembangunan Nasional (UPN) Veteran Jakarta, Jakarta, Special Capital Region of Jakarta, Indonesia; 3 Family Medicine and Primary Care Department, Universitas Pelita Harapan, Tangerang, Banten, 15811, Indonesia

**Keywords:** Cognitive, enhancer, agent, ginkgo biloba, niche

## Abstract

**1) Introduction:**

Cognitive enhancers, or nootropics, are agents that aim to improve cognitive functions such as memory, attention, and executive processing. This bibliometric study investigates the scientific landscape surrounding Ginkgo biloba as a cognitive enhancer, using data retrieved from the Scopus database and analyzed via Biblioshiny and VOSviewer. This bibliometric study analyzes literature from Scopus to identify key trends, influential authors, and research gaps, providing guidance for future research.

**2) Methods:**

This study employs a literature review methodology to gather data from the Scopus database on Neuroaid, analyzing it using Biblioshiny and VOSviewer software. The focus was on Ginkgo Biloba as a niche-theme cognitive enhancer agent based on Scopus data, using both quantitative and qualitative analyses.

**3) Results and discussion:**

Ginkgo biloba, the ‘maidenhair tree’ from the order Ginkgoales, appeared 290 million years ago. Chinese and Japanese culture has been cultivated for thousands of years. This tree is valued for its resilience and therapeutic properties, often used in traditional medicine for respiratory and blood circulation issues.

**4) Conclusions:**

This bibliometric analysis reveals that Ginkgo biloba remains a focal point in cognitive enhancer research, with increasing scholarly attention over the past decade. Despite its historical and cultural significance, the literature shows fragmented research clusters and limited clinical consensus. The study highlights the need for standardized methodologies, stronger interdisciplinary collaboration, and more rigorous clinical trials to validate Ginkgo biloba’s cognitive benefits. These findings provide a roadmap for future research and underscore the importance of bridging traditional knowledge with modern scientific inquiry. This study was conducted in December 2024.

## Introduction

Cognitive enhancers, also known as nootropics or smart drugs, have garnered significant attention from both the scientific community and popular culture. These substances are designed to improve cognitive functions, such as memory, attention, and problem-solving abilities in both healthy individuals and those experiencing cognitive decline. ^1^ The growing interest in cognitive enhancers is driven by their potential benefits in enhancing mental performance, which can be particularly valuable in competitive environments such as academia and the workplace. ^2^

Despite the promising potential of cognitive enhancers, this field faces several challenges, including the need for rigorous scientific validation, ethical considerations, and regulatory hurdles. ^3^ The current body of literature on cognitive enhancers is vast and diverse, encompassing studies on various pharmacological agents, non-pharmacological interventions, and their effects on different cognitive domains. ^4^ However, there is a lack of a comprehensive bibliometric analysis that systematically reviews and synthesizes this body of research.

This bibliometric study aimed to fill this gap by providing a comprehensive analysis of the existing literature on cognitive enhancers indexed in Scopus. By employing bibliometric methods, this study identified key trends, influential authors, and research gaps in the field. The findings of this study will contribute to a better understanding of the current state of research on cognitive enhancers and inform future research.

The methodology of this study involved the collection and analysis of data from Scopus, focusing on articles published between 1988 and 2024. The data will be analyzed using various bibliometric techniques, including co-citation, keyword, and network analyses. The results are presented in the form of visualizations and descriptive statistics, providing a clear and comprehensive overview of the research landscape.

The significance of this study lies in its potential to inform policy decisions, guide research funding and shape the development of cognitive enhancers. By identifying the most impactful studies and emerging trends, this bibliometric analysis will help researchers and policymakers prioritize areas of research that are likely to yield the greatest benefits.

## Methods

### Bibliometric research approach

Bibliometric research employs structured analysis of scientific publishing data to map the evolution of specific disciplines. This study focuses on the topic of cognitive enhancers, aiming to identify trends, patterns, and correlations within academic literature. To ensure transparency and reproducibility, we systematically documented the data collection, search strategy, and analytical procedures.

### Data source and search strategy

This study utilized Scopus ( www.scopus.com), a widely recognized and reputable database for scientific literature. The search was conducted in December 2024 using the following parameters:

• Search Keywords: “cognitive enhancer”
• Search Fields: Title, Abstract, Keywords (TITLE-ABS-KEY)

To ensure comprehensive coverage, no additional filters (such as publication year, language, or document type) were applied. The decision to maintain an unfiltered search was based on the goal of retrieving all relevant publications without introducing potential bias in the selection process.

### Data retrieval and export formats

The retrieved results were exported from Scopus in three distinct formats for bibliometric analysis:

1. CSV (Comma-Separated Values): Provides metadata, including title, authors, affiliations, publication year, source, abstract, and keywords.
2. RIS (Research Information System): Contains detailed bibliographic records, including citation data.
3. TXT (Plain Text): Includes structural elements necessary for further processing in Biblioshiny and VOSviewer.

### Data collection and processing

A search query was executed using “cognitive enhancer” in the TITLE-ABS-KEY field. This resulted in the retrieval of 1420 relevant documents. These documents were exported and stored in .csv format for further bibliometric analysis. The exported data was then processed using Biblioshiny and VOSviewer, which enabled visualization of research trends, co-authorship networks, and keyword clustering.

### Ensuring reproducibility

To enhance reproducibility:

• The search query string (TITLE-ABS-KEY (“cognitive enhancer”)) and data export settings have been clearly documented.
• No additional filters were applied, and justification for this approach is explicitly stated.
• The raw Scopus data exports will be made available upon request to facilitate independent verification.

### Data analysis

Both the Biblioshiny and Vosviewer software packages were utilised in the analysis process.

### Quantitative analysis

Documents by Year

**Figure 1.**
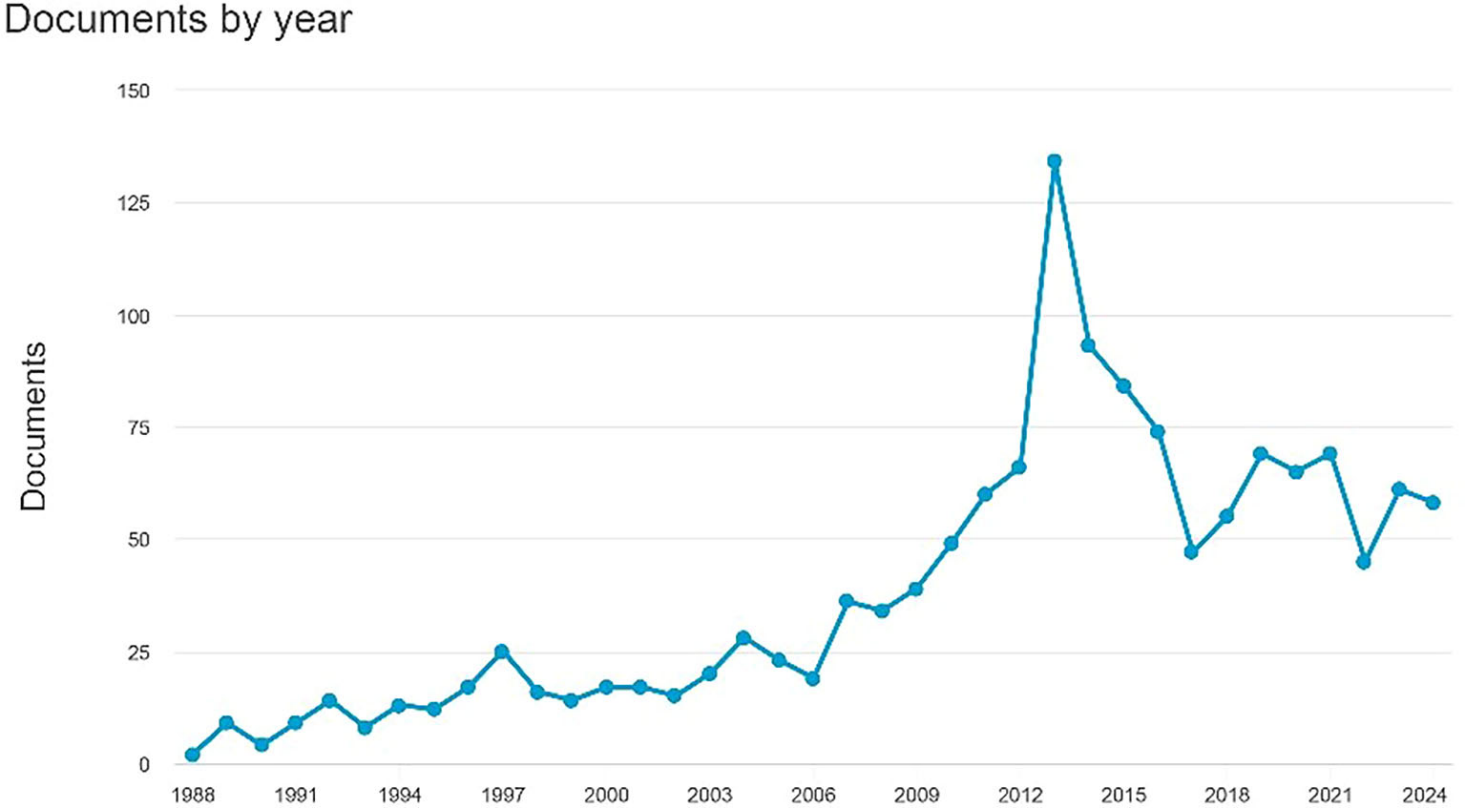
Documents by year.

Figure 1 indicates an increase in the number of documents, culminating in 130 papers by 2014. The earliest document dates back to 1988 and comprises two papers titled “Antagonism by Exifone, a New Cognitive Enhancing Agent, of the Amnesias Induced by Four Benzodiazepines in Mice,” authored by Porsolt, R.D., Lenègre, A., Avril, I., and Doumont, G. The second document, headed “Comparison of the Effects of Vinpocetine, Vincamine, and Nicergoline on the Normal and Hypoxia-Damaged Learning Process in Spontaneously Hypertensive Rats,” is authored by Groó, D., Pálosi, É., and Szporny, L. As of 2024, there are 54 documents, with the most recent titled “MSK 1,” which is essential for the experience- and ampakine-dependent strengthening of spatial reference memory and reversal learning, as well as for the induction of Arc and BDNF written by Morè et al. ^7^

**Figure 2.**
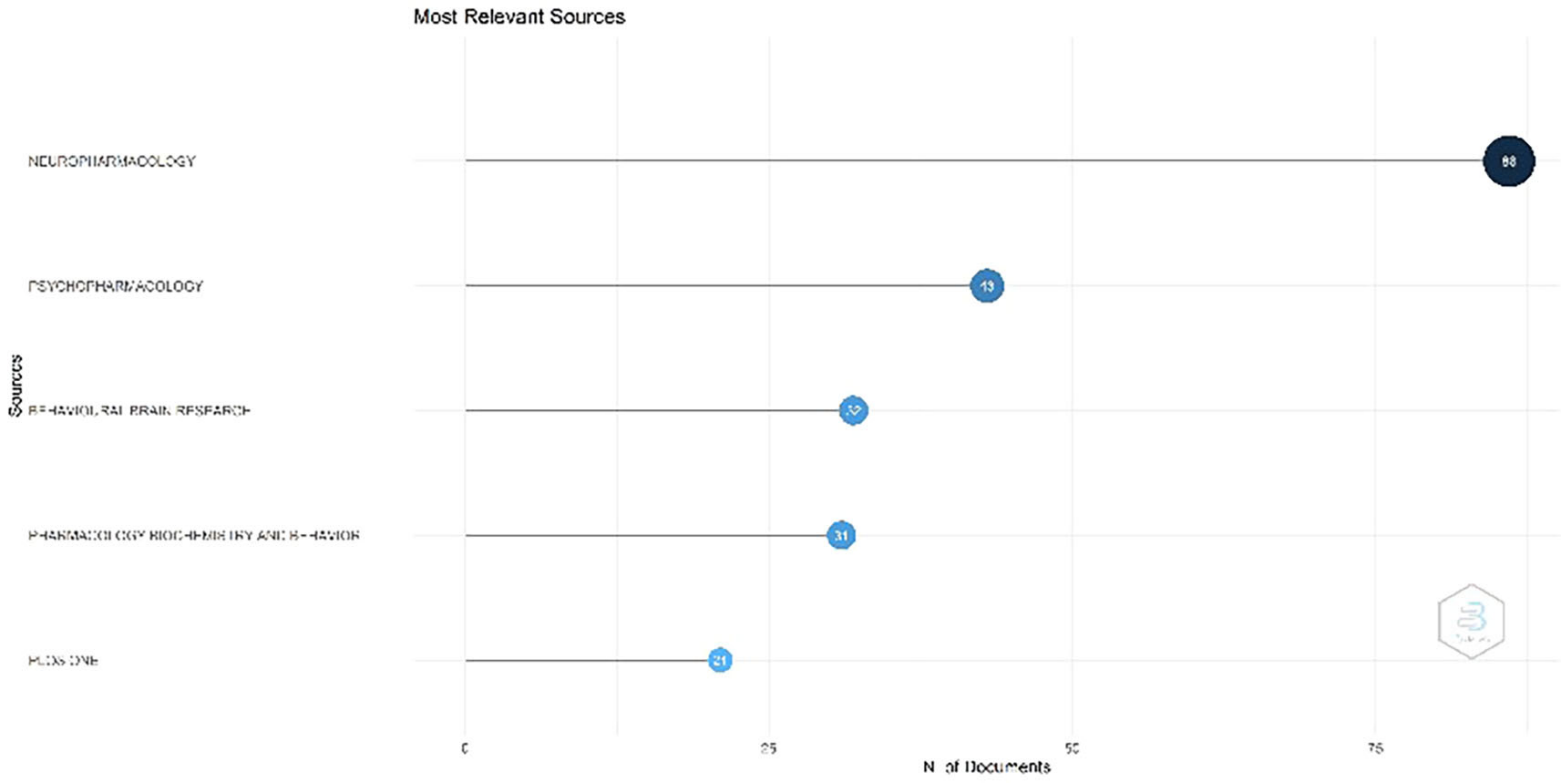
Most Relevant Sources.

Most Relevant Sources

According to Figure 2, Neuropharmacology occupies the foremost position. This contains information regarding the journal neuropharmacology. The journal has been indexed in Scopus since 1962. The current SCImago Journal Rank (SJR) for this journal was 1.307. It was published by Elsevier Ltd. The journal publishes high-quality original research in neuroscience, concentrating on the examination and comprehension of the effects of exogenous and endogenous chemical agents on neurobiological processes in the mammalian nervous system. This journal is classified as Q1 in the fields of cellular and molecular neuroscience, and pharmacology. Psychopharmacology has been indexed in Scopus since 1959. The most recent SJR is 1.053. SCImago quartile classification, Q1 (highest). This magazine accepts many manuscripts, including experimental studies on the impact of medications on human cognition and behavior as well as laboratory investigations involving experimental animals. It also invites works that incorporate various levels of analysis, ranging from neurochemical assays to functional neuroimaging research publishes around 250–300 articles annually across diverse topics in behavioral, clinical, and neuropharmacology, with a moderately selective acceptance rate and broad acceptance of article types including original research, reviews, and methodological studies. Ranking three is the Journal of Behavioral Brain Research, published by Elsevier and indexed in Scopus since 1980, holds a 2024 SJR of 0.919 (Q2), publishes a high volume of ~400–500 articles annually, accepts a broad range of topics in behavioral neuroscience—including neurophysiology, neuropharmacology, neuroimaging, and neuroethology—and while its acceptance rate is moderately selective, its interdisciplinary scope allows for diverse article types such as original research, methodological advances, and critical reviews.

**Figure 3.**
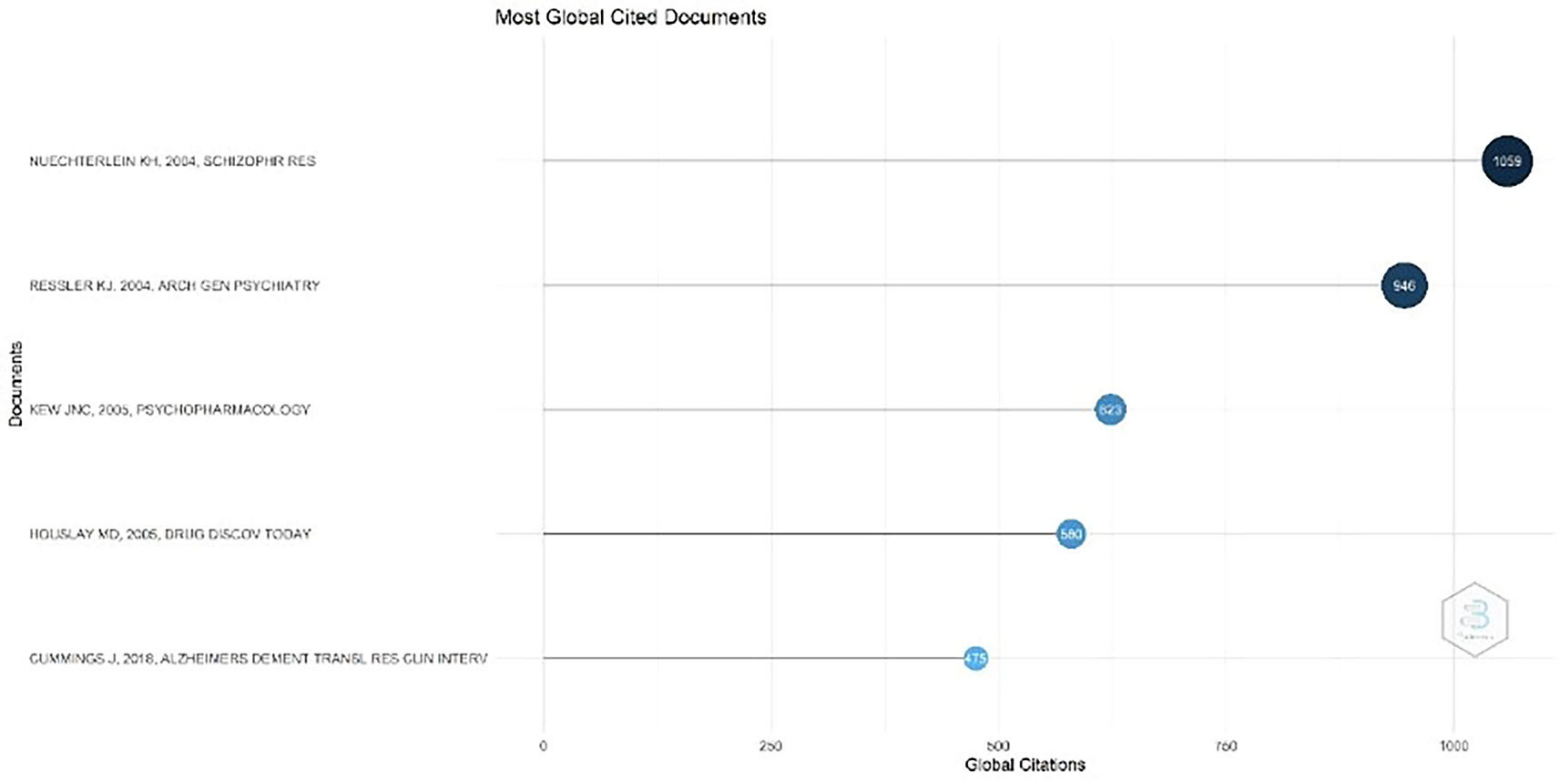
Most Global Cited Documents.

Most Global Cited Documents

According to Figure 3, the most cited document, with 1059 citations, is the article titled Identification of separable cognitive factors in schizophrenia, authored by Keith H. Nuechterlein, Deanna M. Barch, James M. Gold, Terry E. Goldberg, Michael F. Green, and Robert K. Heaton. The journal’s abstract states that one of the main objectives of this research was to identify separate cognitive factors in schizophrenia. This study aimed to better understand how this disorder affects various cognitive aspects, such as memory ability, attention, and information processing.

Second, with 946 citations, a document titled “Cognitive Enhancers as Adjuncts to Psychotherapy: Use of D-Cycloserine in Phobic Individuals to Facilitate Extinction of Fear” was authored by Kerry J. Ressler, MD, PhD; Barbara O. Rothbaum, PhD; Libby Tannenbaum, PhD; and colleagues. Background: This study evaluated the use of D-cycloserine (DCS), a partial agonist of N-methyl-D-aspartate (NMDA) receptors, as an adjunct to exposure therapy for individuals with phobias. Previous studies have demonstrated that DCS can accelerate fear extinction in animals.

The third-ranked document, with 623 citations, is titled Ionotropic and Metabotropic Glutamate Receptor Structure and Pharmacology. This review was published on February 25, 2005, in Psychopharmacology. The authors of this review were James N. C. Kew and John A. Kemp. This document discusses ionotropic and metabotropic glutamate receptors, which are two major receptor types that mediate the actions of glutamate—the primary excitatory neurotransmitter in the central nervous system (CNS).

Factorial Map Of The Documents With The Highest Contributes

**Figure 4.**
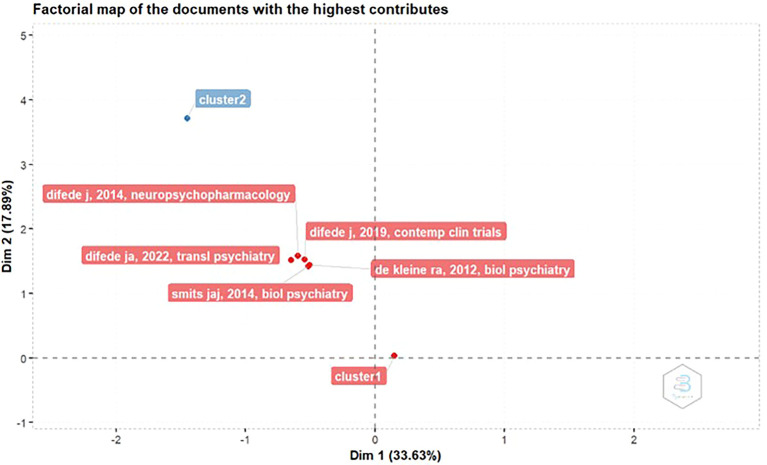
Factorial map of the documents with the highest contributes.

In Figure 4, this figure presents a factorial map derived from principal component analysis (PCA), visualizing the distribution of studies based on their contributions to the first two principal dimensions: **Dim 1 (33.63%)** and **Dim 2 (17.89%)**, which together explain 51.52% of the total variance in the dataset. *Dim 1* primarily captures variance related to study design features and therapeutic modality, while *Dim 2* reflects variance associated with publication year and neuropharmacological intervention type.

The red-labeled points represent individual studies with the highest contributions to these dimensions. Notably, the manuscript by **Difede et al. (2014)** titled *“D-Cycloserine Augmentation of Exposure Therapy for Post-Traumatic Stress Disorder: A Pilot Randomized Clinical Trial”* (published online in *Neuropsychopharmacology* on December 11, 2013) shows the strongest contribution to Dim 1, indicating its central role in shaping the variance structure of the dataset. Although the study was published online in 2013, its formal citation year is 2014, which explains the labeling discrepancy noted by the reviewer.

The labels **cluster 1** and **cluster 2** refer to centroids derived from hierarchical clustering applied to the PCA coordinates. While each centroid appears as a single point, they represent the mean position of multiple studies grouped by similarity in PCA space. However, the visual proximity of several studies—such as those by Difede (2014, 2019), Smits (2014), and de Kleine (2012)—suggests a natural grouping based on shared methodological or pharmacological features. This emergent grouping is not explicitly labeled as a cluster in the figure but is discussed here to clarify the underlying structure.

This visualization aids in identifying which studies most influence the dimensional structure of the dataset and highlights thematic convergence among trials investigating D-cycloserine augmentation in PTSD treatment.

Documents by Author

**Figure 5.**
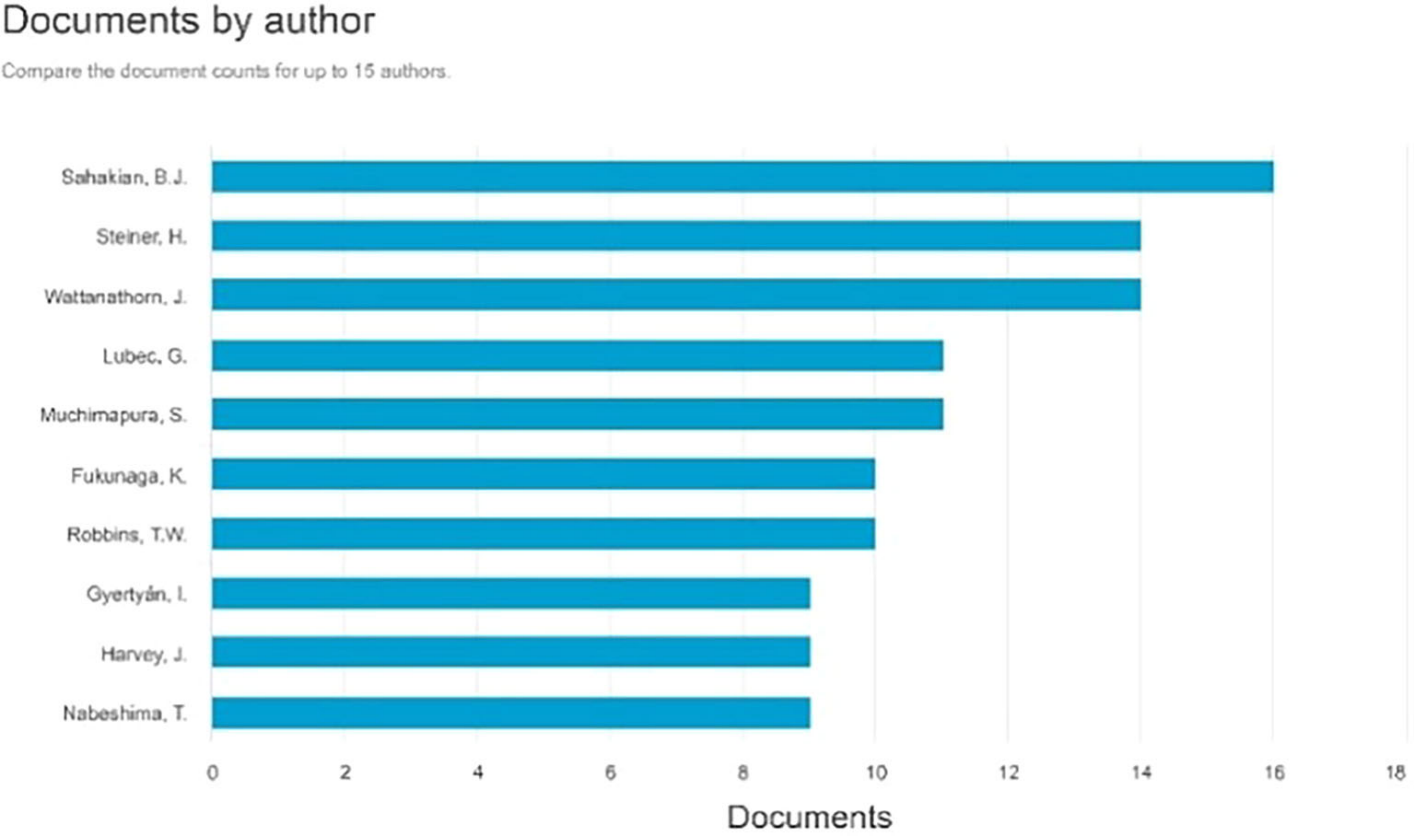
Documents by Author.

According to Figure 5, the three most prolific authors, each with 16 documents, are as follows: Sahakian, B.J., with many paper titles: Enhancement of the motor control network with methylphenidate in individuals with traumatic brain injury. Effects of atomoxetine on attentional bias towards drug-related signals in cocaine-dependent individuals Modafinil Enhances Episodic and Working Memory Cognition in Patients with Remitted Depression: A Double-Blind, Randomized, Placebo-Controlled Study. Examining the Neuroscience and Societal Consequences of Cognitive Enhancers as well as the utilization of substances by healthy individuals to augment cognition, creativity, motivation, and enjoyment. ^8– 12^

The subsequent author with 14 publications is Steiner, H., whose articles include: Methylphenidate with or without fluoxetine triggers the restoration of cocaine-seeking behavior in rats. Vilazodone, an innovative SSRI antidepressant with 5-HT1A partial agonist characteristics, reduced the potentiation of chronic oral methylphenidate-induced dynorphin expression in the striatum of male adolescent rats. Fluoxetine amplifies the behavioral effects generated by methylphenidate: increased locomotion or stereotypies, and improved acquisition of cocaine self-administration. Concurrent Chronic Administration of Oral Methylphenidate and Fluoxetine During Adolescence: Behavioral Effects, and Fluoxetine Enhances Oral Methylphenidate-Induced Gene Regulation in the Rat Striatum. ^13– 17^

The subsequent author with 14 publications is Wattanathorn, J., whose articles include: Effect of Single Administration of Mulberry Milk on the Cognitive Function of 6-12-Year-Old Children: Results from a Randomized, Placebo-Controlled, Crossover Study. Assessment of the neuroprotective and cognitive enhancement properties of Cucurbita moschata. Mung bean-derived protein protects against neurodegeneration and memory deficits in an animal model of menopause with obesity. The amalgamated extract of black sticky rice and dill enhances cognitive deficits following stroke in individuals with metabolic syndrome and functional beverages. A novel cognitive enhancer, comprising extracts of purple corn cob and pandan leaves, enhances spatial memory and hippocampal neuron density by improving extracellular signal-regulated protein kinase expression, cholinergic function, and oxidative status in ovariectomized rats. ^18– 22^

Documents by country or territory

**Figure 6.**
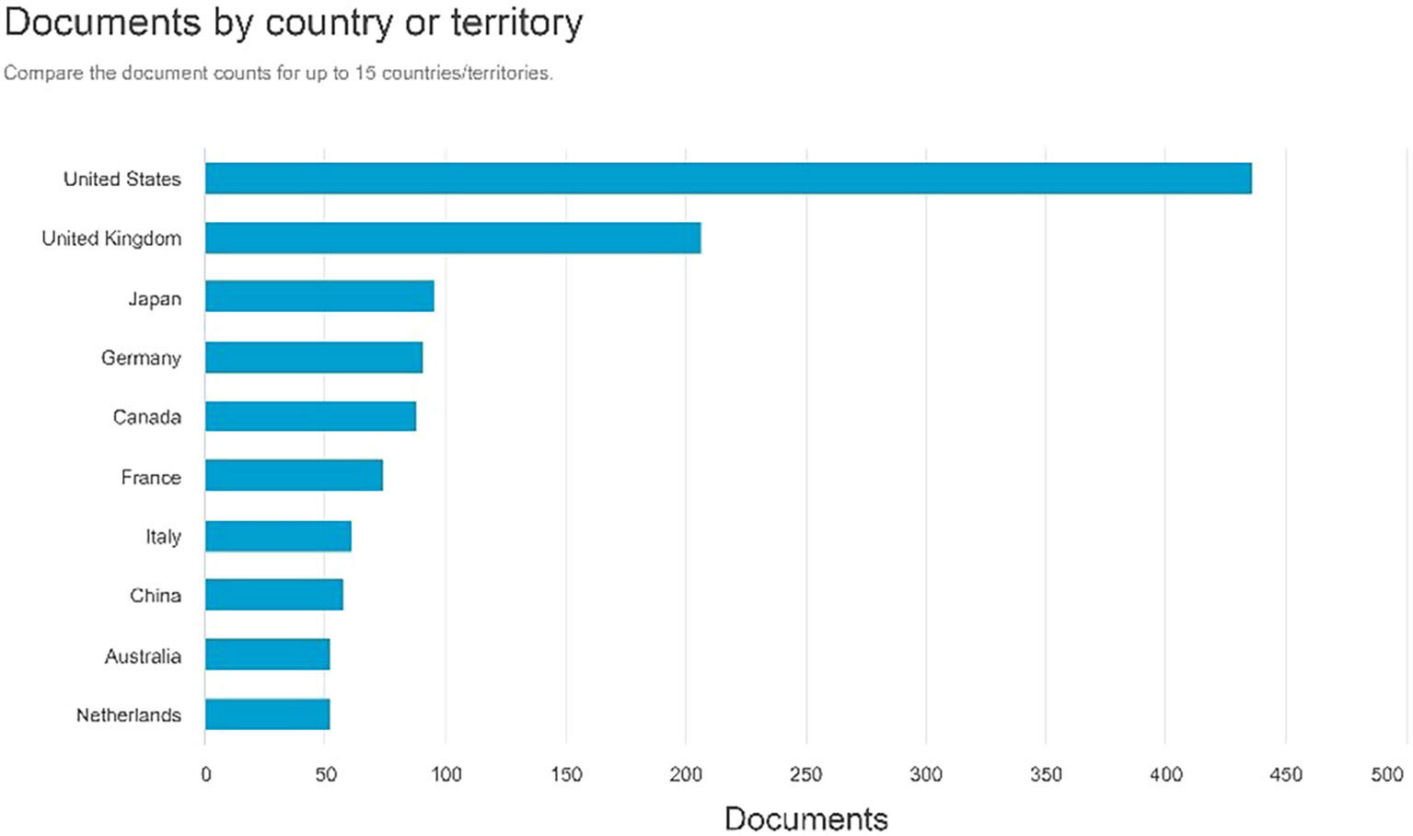
Documents by country or territory.

Figure 6 indicates that the United States has the highest number of document creators, totaling 436 documents. The United Kingdom followed 206 documents, followed by Japan with 95. Germany possesses 90 documents, whereas Canada has 87.

Documents by Subject Area

**Figure 7.**
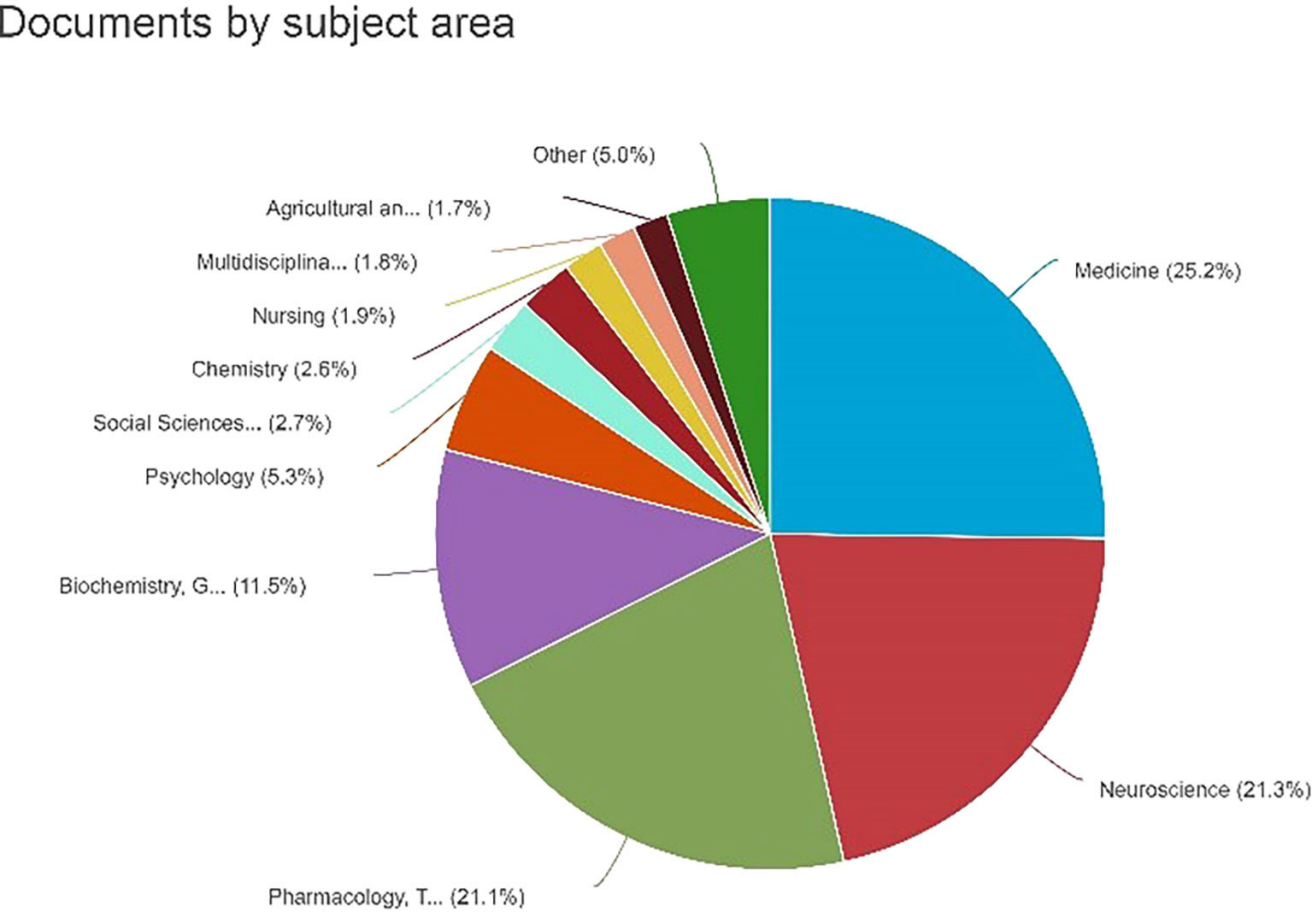
Documents by Subject Area.

Figure 7 shows a pie chart depicting the distribution of the documents by subject area. The graph shows the percentage of documents classified into different subject areas. The percentage details for each topic area were as follows: medicine: 25.2% or 614 documents. Neuroscience: 21.3% (518 documents) Pharmacology, Toxicology, and Pharmaceutics: 21.1% (513 documents) Biochemistry, Genetics, and Molecular Biology: 11.5% (280 documents) Psychology: 6.3% or 280 documents. Social Sciences: 2.7% or 65 documents. Chemistry: 2.6% or 64 documents. Nursing: 1.9% or 47 documents. Multidisciplinary: 1.8% or 44 documents. Agricultural and Biological Sciences: 1.7% or 41 documents, which is noteworthy as it illustrates the substantial dispersion of documents across several topic areas, with medicine comprising the largest percentage. It is crucial to comprehend the study or publication emphasis in various fields.

Documents by affiliation

**Figure 8.**
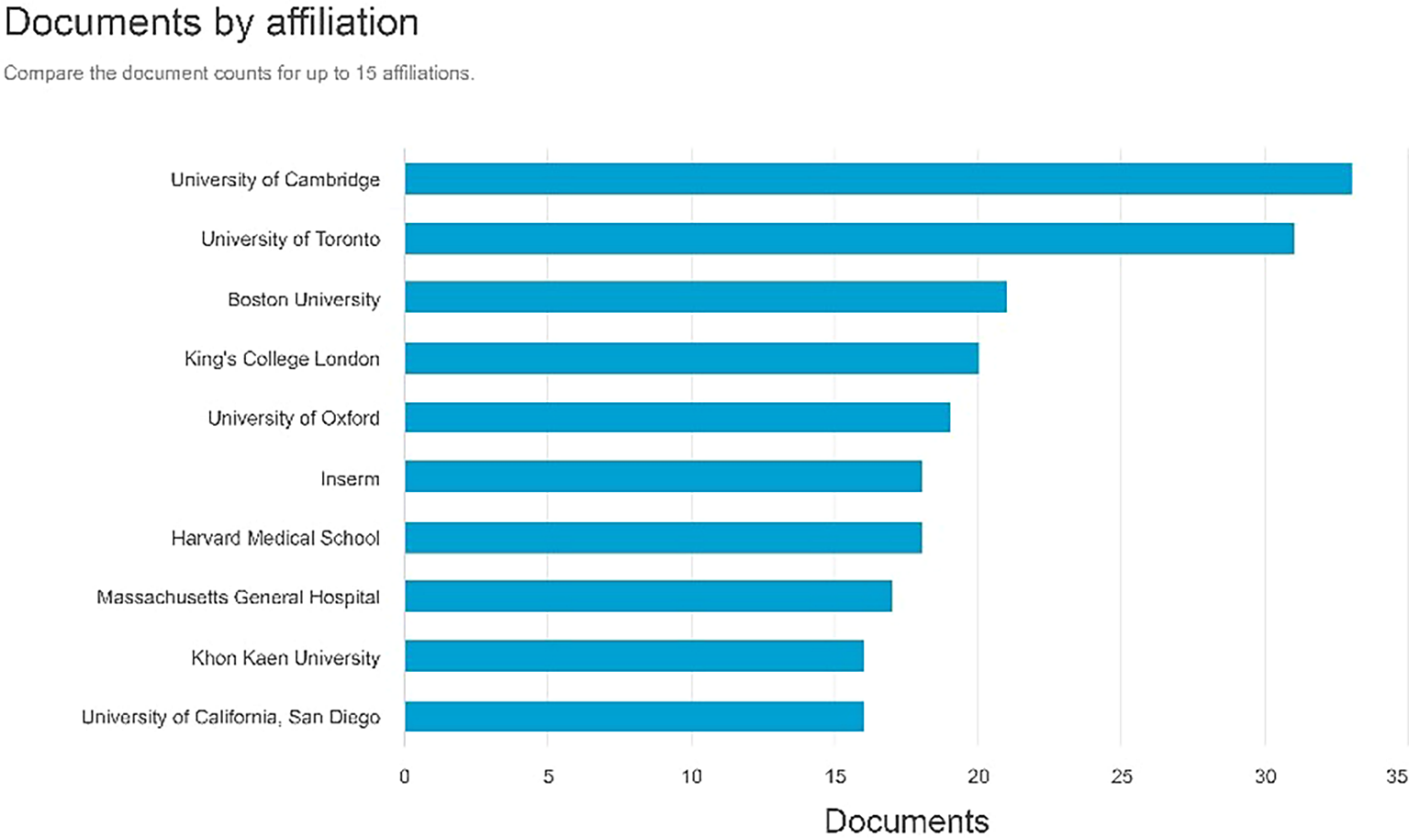
Documents by affiliation.

According to Figure 8, in first place, the producer of most documents is affiliated with the University of Cambridge with 33 documents, the second is affiliated with the University of Cambridge with 31 documents, and the Boston University with 21 documents.

Documents by funding sponsor

**Figure 9.**
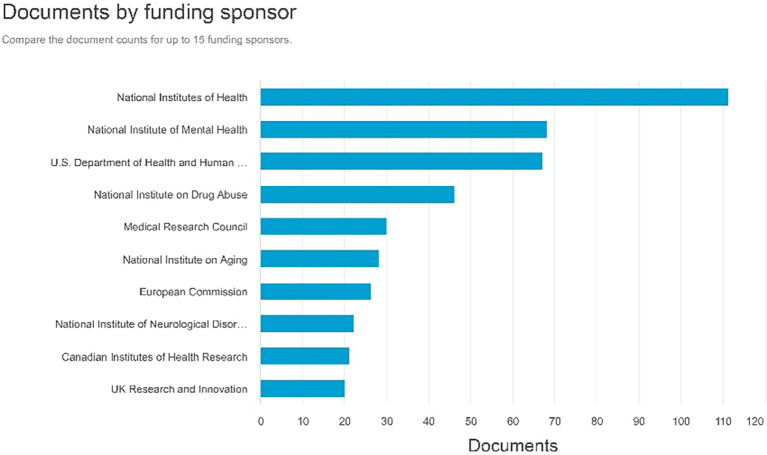
Documents by funding sponsor.

Referring to Figure 9, the sponsors that generate the highest number of documents for funding are the National Institutes of Health with 111 documents, followed by the National Institute of Mental Health with 68 documents, and the U.S. Department of Health and Human Services producing 67 documents.

**Figure 10.**
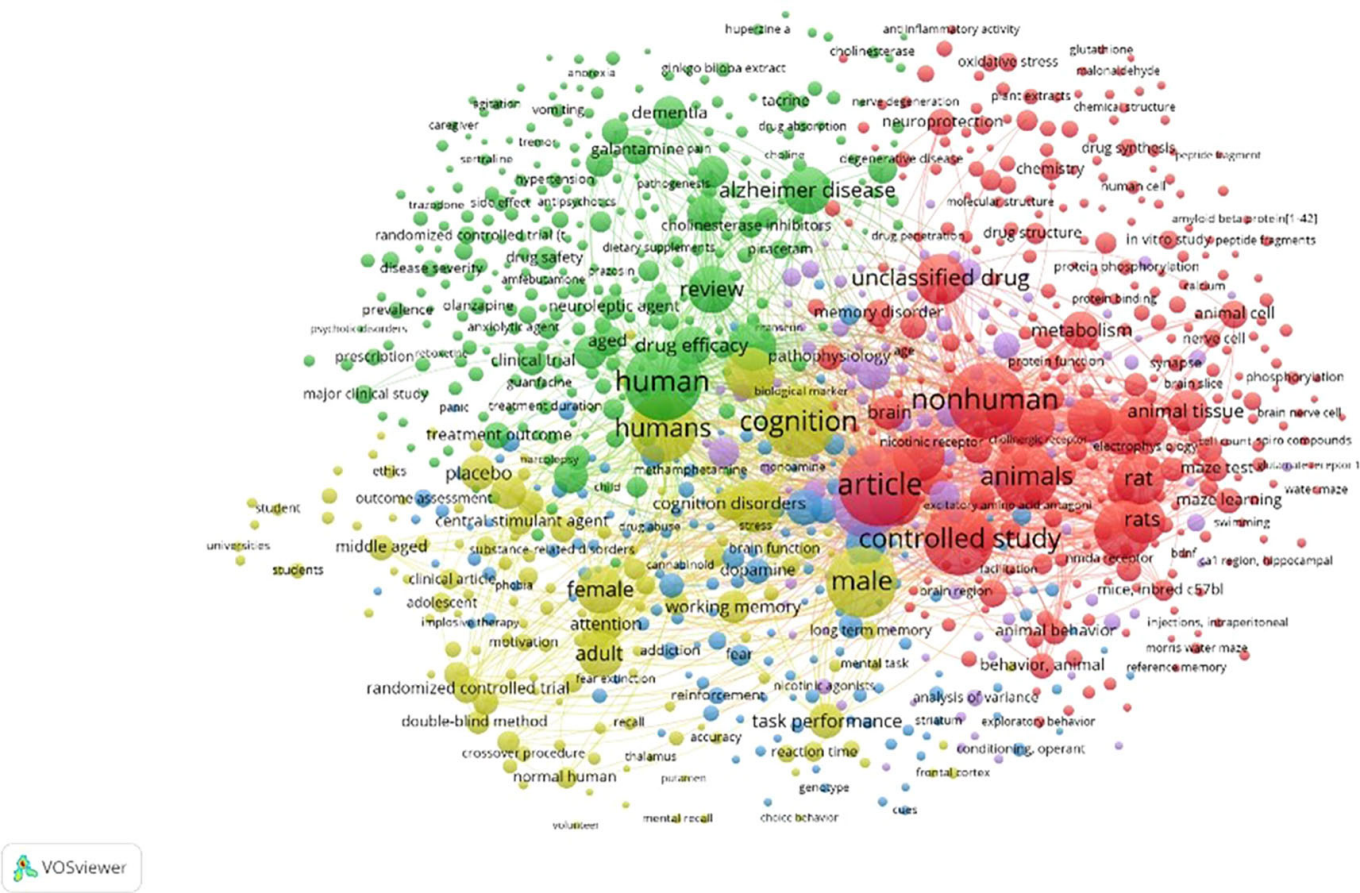
Network Visualization.

Network Visualization

Figure 10, It can be seen that the area being studied is still not connected to other areas. The domain encompasses agitation, anorexia, vomiting, caregiver, tremor, hypertension, dietary supplement, Ginkgo biloba extract, huperzine A, anti-inflammatory activity, oxidative stress, glutathione, malondialdehyde, plant extracts, chemical structure, drug synthesis, peptide fragment, amyloid beta-protein, ^1– 42^ in vitro studies, peptide fragments, protein, phosphorylation, calpain, animal, cell, nerve cell, phosphorylation, brain nerve cell, compounds, water maze, NMDA receptor 1, hippocampal CA1 region, BDNF, swimming, Morris water maze, reference memory, cues, choice behavior, mental recall, volunteer, normal human, crossover, procedure, double-blind method, randomized controlled trial, adolescent, impulsive behavior, phobia, substance-related disorders, clinical article, ethics, outcome assessment, universities, students, and students.

Overlay Visualization of Scopus, Database Using Vosviewer

**Figure 11.**
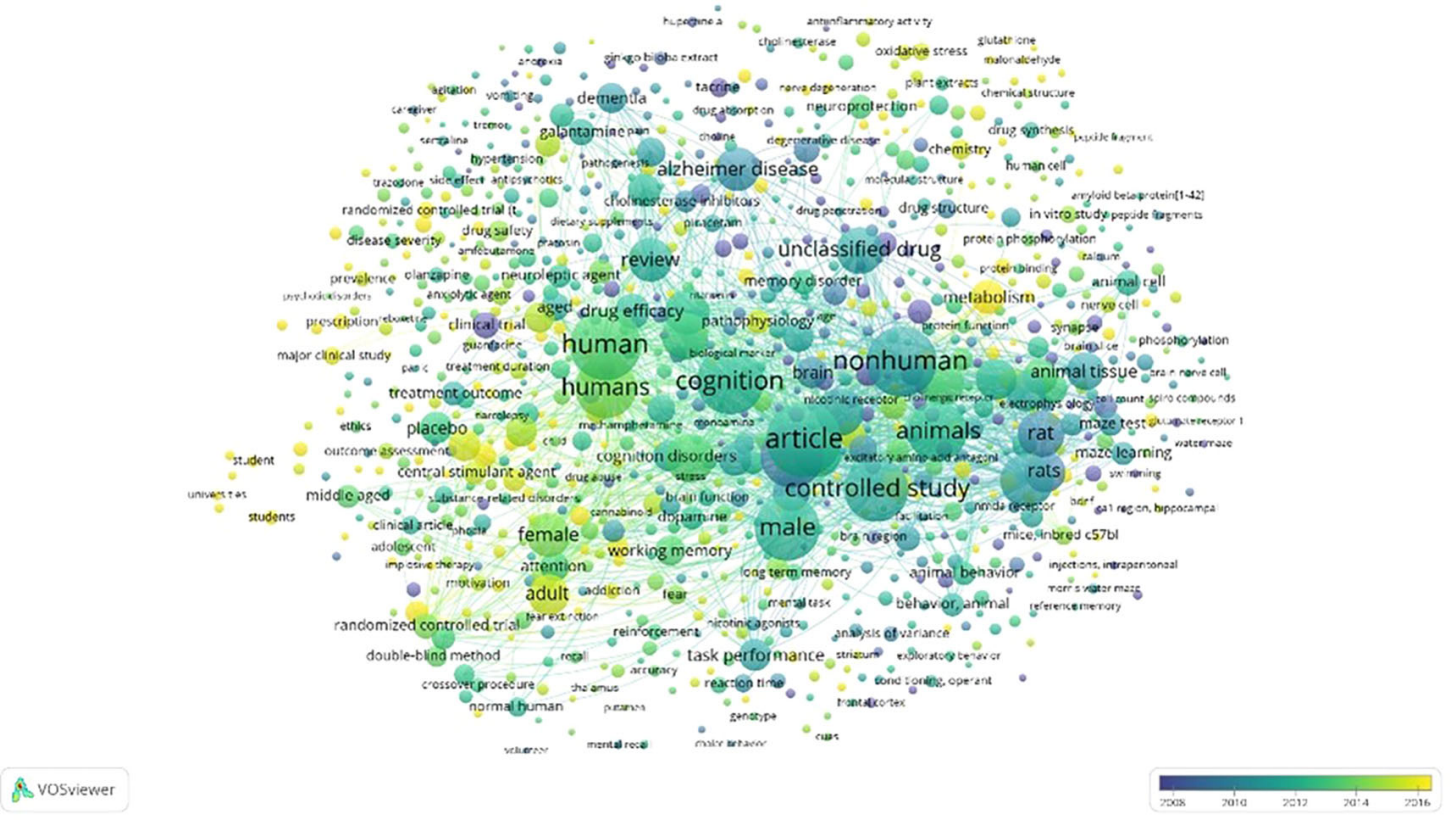
Overlay visualization of scopus, database using Vosviewer.

According to Figure 11, in the overlay visualization, it appears that the keywords that are being researched a lot approaching 2016 are the parts colored yellow, namely gluthahione, malonaldehyde, metabolism, prescription, major clinical study, randomized controlled trial (t, addiction, student, students, universities, oxidative stress, chemistry, and nerve degeneration.

Density Visualization

**Figure 12.**
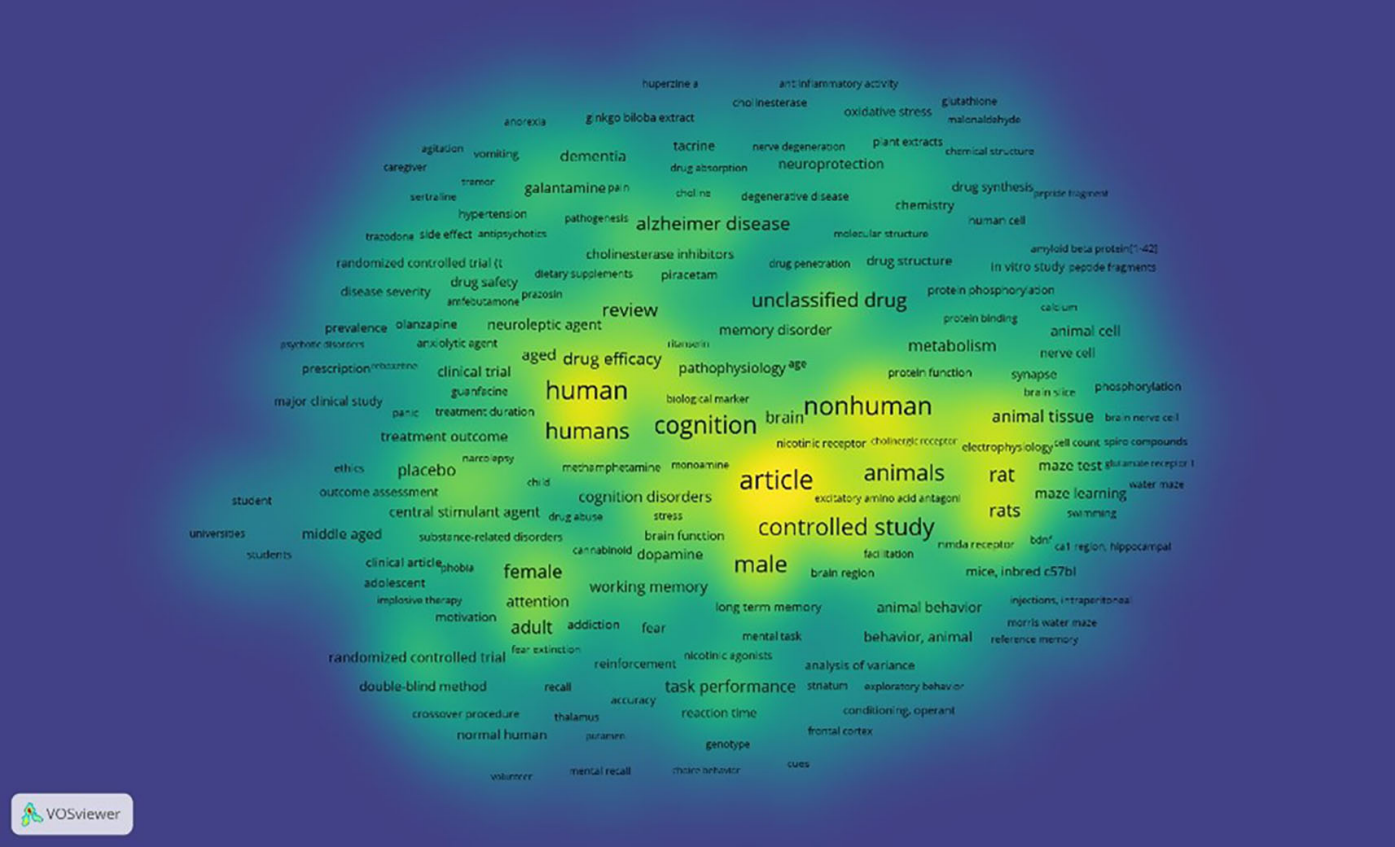
Density visualization.

This is illustrated in Figure 12, in the visual circulation density, it appears that the part that is already saturated with research is yellow, while the part that is not yet saturated is slightly yellow and dominantly green, namely keywords: agitation, vomiting, anorexia, ginkgo biloba extract, cholinesterase, oxidative stress, glutathione, malonaldehyde, galantamine, pain, dementia, tacrine, nerve degeneration, plant extracts, sertraline, tremor, hypertension, neuroprotection, choline, degenerative disease, trazodone, side effect, antipsychotics, pathogenesis, alzheimer disease, cholinesterase inhibitors, dietary supplements, piracetam, drug penetration, drug structure, randomized controlled trial (t, drug safety, amfebutamone, prazosin, protein phosphorylation, peptide fragments, prevalence, olanzapine, neuroleptic agent, anxiolytic agent, ritanserin, memory disorder, amyloid beta protein[1-42], psychotic disorders, prescription, reboxetine, clinical trial, guanfacine, drug synthesis, chemistry, molecular structure, drug structure, in vitro study, peptide fragments, treatment outcome, narcolepsy, cell count, spiro compounds, ethics, placebo, outcome assessment, central stimulant agent, drug abuse, stress, universities, major clinical study, middle aged, substance-related disorders, clinical article, adolescent, phobia, impulsive therapy, motivation, attention, adult, addiction, fear, reinforcement, recall, accuracy, normal human, volunteer, randomized controlled trial, double-blind method, crossover procedure, mental recall, choice behavior, brain function, dopamine, task performance, reaction time, conditioning, operant, frontal cortex, genotype, mental recall, choice behavior, facilitation, nmda receptor, bdnf, ca1 region, hippocampal, mice, inbred c57bl, injections, intraperitoneal, animal behavior, behavior, animal, analysis of variance, striatum, exploratory behavior, morris water maze, reference memory, swimming, water maze.

Thematic Map

**Figure 13.**
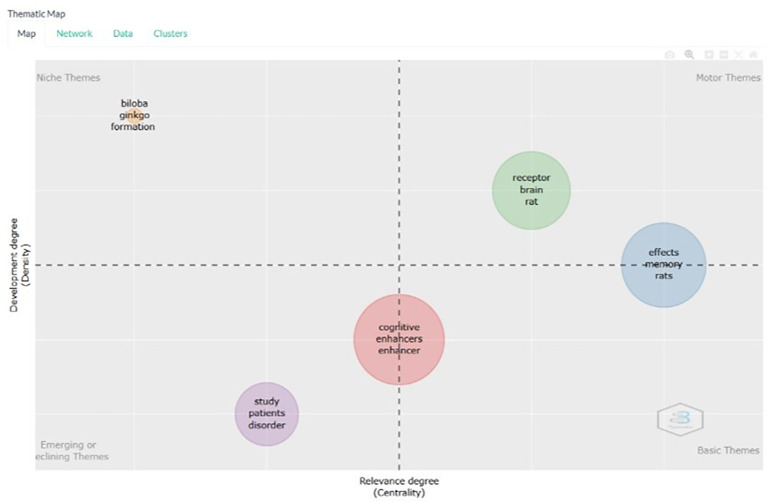
Thematic map.

According to Figure 13, on the thematic map based on the title, the following is an explanation for each keyword in each quadrant in the thematic map resulting from the bibliometric analysis. Here is an explanation of the meaning of each quadrant in the thematic map, and examples of document titles relevant to keywords in each quadrant.

Figure 13 illustrates a thematic map generated from bibliometric analysis of document titles. The map is divided into four quadrants, each representing a distinct category of research development based on two dimensions: density (indicating internal development) and centrality (indicating relevance to the broader field). Each quadrant contains clusters of keywords that reflect the thematic focus and maturity of research topics.

Niche Themes (Upper-Left Quadrant):

• These are specialized, well-developed topics with limited influence on the broader research field. Keywords such as *biloba*, *ginkgo*, and *formation* suggest focused investigations into natural compounds. Example titles include: *Neuroprotective effects of Ginkgo biloba in Alzheimer’s disease* and *Formation of cognitive enhancers from natural sources: The case of Ginkgo biloba*.

Motor Themes (Upper-Right Quadrant):

• These are both well-developed and central to the field, representing the driving forces of current research. Keywords like *receptor*, *brain*, *rat*, *effects*, and *memory* indicate robust experimental studies. Example titles include: *Role of NMDA receptors in brain function and cognition* and *Effects of novel cognitive enhancers on memory formation in rats*.

Basic Themes (Lower-Right Quadrant):

• These are foundational topics that are central but not yet fully developed. Keywords such as *cognitive*, *enhancer*, and *enhancers* point to broad conceptual frameworks. Example titles include: *Overview of cognitive enhancers: mechanisms and applications* and *Cognitive enhancers: A review of their efficacy and safety*.

Emerging or Declining Themes (Lower-Left Quadrant):

• These represent areas that are either gaining traction or losing relevance. Keywords like *study*, *patients*, and *disorder* suggest clinical applications and evolving interest. Example titles include: “ *Clinical studies of cognitive enhancers in patients with cognitive disorders* and *Emerging trends in cognitive enhancer research: a clinical perspective*.”

Thematic Evolution

**Figure 14.**
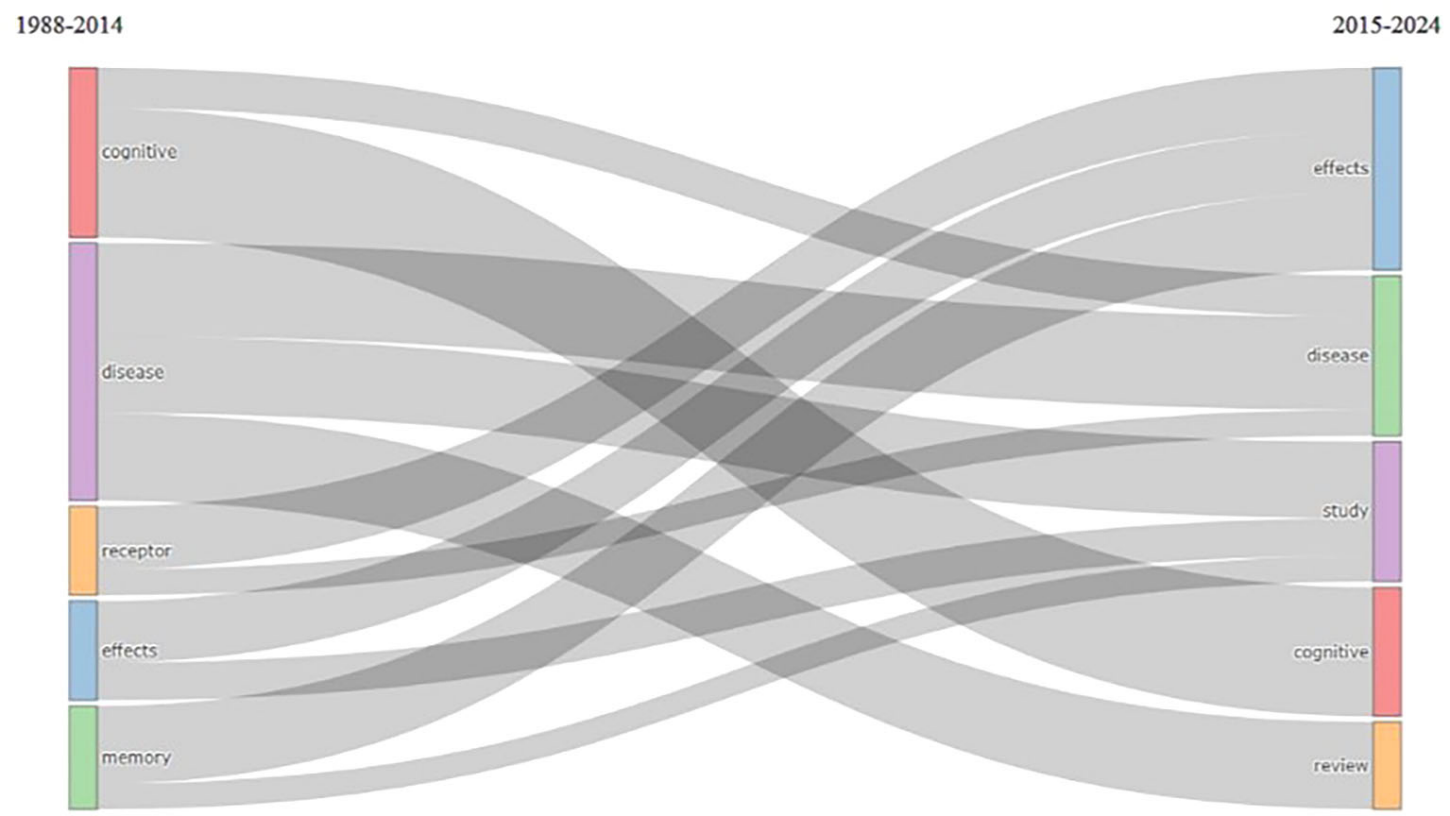
Thematic Evolution.

According to Figure 14, there was an evolution of changes in themes in research from 1988 to 2014 with the keywords cognitive, disease, receptor, effects, and memory. The theme then changed in 2015–2024 to effects, disease, study, cognition, and review.

Topic Dendogram

**Figure 15.**
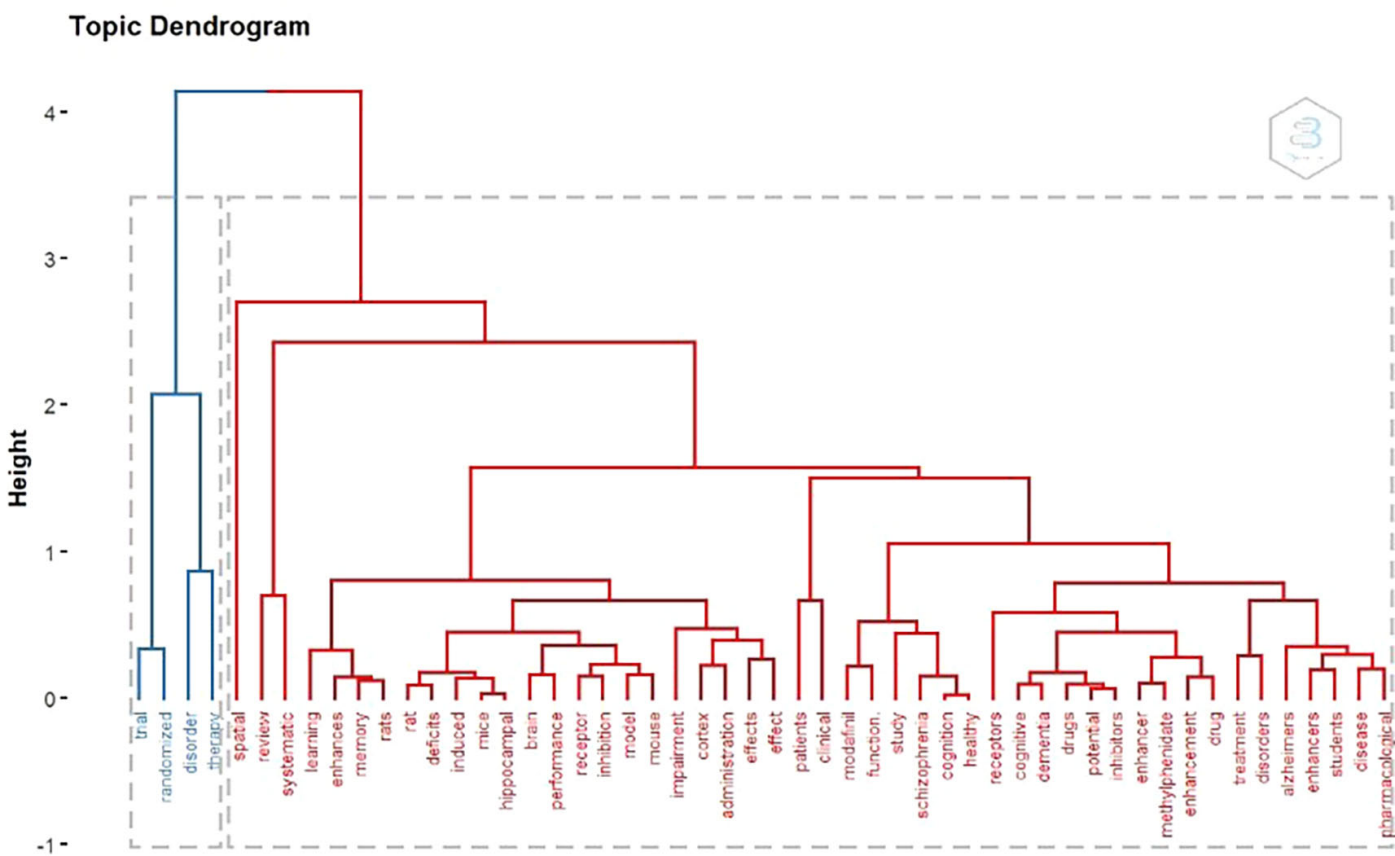
Dendogram.

According to Figure 15, there are 2 large clusters According to keywords. There are 2 clusters of blue and red.

**Qualitative Analysis - refer to extended data for table 1** ^1420^

## Results and Discussion

Ginkgo biloba, also known as the maidenhair tree, is a living species of the order Ginkgoales, which first appeared over 290 million years ago. This plant has been known and developed by Chinese and Japanese cultures for thousands of years, and is often planted in temple gardens. ^1415^ This tree is valued not only for its resilience, but also for its therapeutic properties, which have been utilized in traditional medicine for centuries. In ancient China, Ginkgo biloba leaves have been used in medicinal practices to address respiratory disorders and improve blood circulation. ^1416^

The use of G. biloba as medicine has been documented in various ancient medical texts. In traditional Chinese medicine, ginkgo leaves are often made into tea or liquid extracts for use as a tonic to improve blood flow and address digestive and respiratory issues. Over time, the use of G. biloba has spread to various cultures worldwide, including Japan and Korea, where it continues to be valued for its health benefits. ^1417^

With advancements in pharmaceutical technology, G. biloba extracts have been formulated more efficiently to maximize their therapeutic benefits. The most commonly known standardized extract is EGb 761, which contains 24% flavonoid glycosides and 6% terpenoids. This formulation ensures consistency and product quality. The extract is used in various supplements including tablets, capsules, and liquids. ^1418^

The usual dosage for G. biloba supplements is 40 mg three times a day or 80 mg twice a day, with a maximum dose not exceeding 240 mg/day. This dosage is based on research showing the effectiveness and safety of these doses in improving cognitive function and reducing the symptoms associated with cognitive disorders. Studies have also indicated that proper dosing can have significant effects without causing harmful side effects. ^1419^

Ginkgo biloba has various complex mechanisms. One of the primary mechanisms is the enhancement of blood flow, especially to the brain, through its vasodilator effect. This helps improve oxygenation and nutrient delivery to brain cells, which in turn can enhance cognitive function. Additionally, G. biloba contains flavonoids and terpenoids with antioxidant properties, which help protect brain cells from oxidative damage caused by free radicals.

In addition to enhancing blood flow, flavonoids in G. biloba also have strong antioxidant effects. These antioxidants scavenge free radicals, which can damage cells and tissues in the body. On the other hand, terpenoids have anti-inflammatory properties that help reduce inflammation, which can cause tissue damage and cognitive disorders. The combination of antioxidant and anti-inflammatory effects makes G. biloba an effective therapeutic agent for protecting and improving brain function.

In the modern era, G. biloba has become the subject of various clinical studies investigating its effectiveness in various health conditions. One of the most prominent areas of research is its use in the treatment of dementia and mild cognitive impairment. Studies have shown that G. biloba extract can help improve memory, attention, and overall cognitive function in individuals with mild-to-moderate cognitive impairment.

Although G. biloba is generally considered safe, some side effects have been reported, including headache, dizziness, digestive issues, and allergic reactions. Therefore, it is important to follow the recommended dosage and consult with a healthcare professional before starting Ginkgo biloba supplements, especially for individuals who are taking other medications or have certain health conditions.

Some Ginkgo biloba products available on the market include Cereton, Cerviboost, Gingkan, Ginkgo Biloba Capsule, and GNC Ginkgo Biloba Plus. These products are available in various forms including tablets, capsules, and syrups. Each product typically lists the standardized content of G. biloba extract, making it easier for consumers to choose according to their needs. ^1419^

Many clinical trials have investigated the effectiveness of G. biloba in various health conditions. These studies included diverse populations, including healthy individuals, patients with dementia, and individuals with mild cognitive impairment. Clinical trials have shown that G. biloba can help improve cognitive function and memory, as well as reduce symptoms of dementia and other cognitive disorders. Dosage standardization of G. biloba has also been carried out to ensure consistency and safety during its use.

Further research has shown that G. biloba can affect neurotransmitters in the brain. For instance, G. biloba extract has been found to increase serotonin and dopamine levels, which play key roles in mood regulation and cognitive function. This indicates that in addition to its vasodilator and antioxidant effects, G. biloba can also influence biochemical pathways in the brain that contribute to improved cognitive function.

Research has shown that G. biloba can provide significant benefits in high-risk populations, such as the elderly and individuals with a genetic predisposition to cognitive disorders. In these populations, the use of G. biloba has been associated with a slower rate of cognitive decline and an overall improvement in quality of life. This underscores the potential of G. biloba as an effective preventive intervention. ^1415^

To ensure the quality and safety of G. biloba products, strict regulations have been established in many countries. Marketed products must meet specific standards regarding the content and purity of the G. biloba extract. This includes standards for the concentration of flavonoid glycosides and terpenoids as well as testing for contaminants that may be present in the final product. ^1417^

Additionally, comparative studies between G. biloba and other cognitive agents, such as donepezil and memantine, have been conducted. These studies aimed to determine the relative effectiveness of G. biloba compared with more commonly used medications in the treatment of dementia and cognitive disorders. The results of these studies indicated that G. biloba has a unique benefit and risk profile, making it an attractive option for use in combination with other therapies.

Although many studies have shown the benefits of G. biloba, there are still challenges in terms of the research methodology and variability of results. Further research is needed to understand the precise mechanisms of action and to determine the optimal dosage for various populations and health conditions. Further efforts are required to enhance the regulation and standardization of Ginkgo biloba products in the global market.

This bibliometric analysis not only reflects the quantitative increase in publications on Ginkgo biloba but also reveals the thematic dynamics and scientific collaboration networks shaping the global research landscape. Frequently occurring keywords such as “Alzheimer’s disease,” “oxidative stress,” and “EGb 761” indicate that Ginkgo biloba’s scientific visibility is strongly influenced by its clinical relevance and neuroprotective potential.

Author and institutional mapping reveals a predominance of multidisciplinary collaborations between neurology, pharmacology, and traditional medicine, particularly from institutions in Europe, North America, and East Asia. This trend indicates that bibliometric influence depends not only on the number of publications but also on cross-disciplinary integration and translational framing in scientific communication.

Furthermore, citation analysis reveals that high-impact articles tend to combine experimental approaches with strong clinical narratives, reinforcing Ginkgo biloba’s position as a therapeutic agent bridging the gap between traditional medicine and modern pharmaceuticals. Despite the rich cultural significance of the ethnobotanical and historical uses of Ginkgo biloba, its contribution to the high-impact literature remains limited, suggesting a research bias toward clinical efficacy and molecular mechanisms.

Thus, this study highlights the importance of a bibliometric approach in understanding how the structure of scientific communication influences the perception and direction of Ginkgo biloba research globally. These findings can inform more balanced, inclusive, and contextualized research strategies, particularly in highlighting contributions from developing countries and local medicinal traditions.

## Conclusions

This bibliometric study highlights the increasing scientific interest in Ginkgo biloba as a cognitive enhancer, demonstrating a steady rise in research outputs from 1988 to 2024. By mapping publication trends, influential studies, and keyword clusters, the analysis provides valuable insights into the scope and trajectory of research in this domain.

While Ginkgo biloba has been widely studied for its vasodilatory, antioxidant, and neuroprotective properties, bibliometric findings do not independently verify clinical effectiveness. The literature suggests promising therapeutic potential, particularly in memory enhancement and neurodegenerative disorders, but methodological inconsistencies in clinical trials underscore the need for standardized research protocols and larger sample sizes.

This study also identifies gaps in comparative research between Ginkgo biloba and established pharmacological treatments such as donepezil and memantine, calling for further investigations to clarify efficacy, safety, and optimal dosing strategies. Additionally, regulatory frameworks remain crucial in ensuring quality control and product standardization across global markets.

Future research should focus on refining experimental designs, integrating systematic clinical trials and conducting long-term safety assessments. By maintaining a clear distinction between bibliometric trends and clinical claims, this study serves as a foundation for guiding future investigations into the cognitive-enhancing potential of Ginkgo biloba.

## Ethics and consent

Ethical approval and consent were not required.

## Reporting guidelines

**Figshare:** PRISMA-ScR checklist and flow chart: Gingko Biloba as a niche theme cognitive enhancer agent, 1420 dokumen of Scopus data Base. A bibliometric studi from 1988 to 2024,

DOI: 10.6084/m9.figshare.28081724 ( https://doi.org/10.6084/m9.figshare.28081724.v1) ^1420^

The project contains the following reporting guidelines:

• PRISMA_2020_checklist_AYS
• PRISMA_2020_flow_diagram_new_AYS

The data are available under the terms of the Creative Commons Attribution 4.0 International license (CC-BY 4.0).

## Software availability

VOSviewer software is an open-access tool that can be used as a cost-effective method for scientometric analysis Biblioshiny

## Author contribution

AYS conducted research, gathered data, performed statistical analysis, and produced discussions and conclusions, including TDS, RV, and DAYS editing.

## Data Availability

No Data Associated with this manuscript. Figshare: Gingko Biloba as a niche theme cognitive enhancer agent, 1420 dokumen of Scopus data Base. A bibliometric studi from 1988 to 2024. DOI: 10.6084/m9.figshare.28081709 ( https://doi.org/10.6084/m9.figshare.28081709.v1) ^1420^ This project contains the following underlying data:
• Density Visualization.jpg • Documents by affiliation.jpg • Documents by Author.jpg • Documents by country or territory.jpg • Documents by funding sponsor.jpg • Documents by Subject Area.jpg • Documents by Year.jpg • Factorial Map Of The Documents With The Highest Contributes.png • Factorial Map Of The most cited documents.png • MostRelevantSources-2024-12-03.png • Network Visualization.jpg • Overlay Visualization of Scopus, Database Using Vosviewer.jpg • tematic evolution berdasarkan title.jpg • Thematic map.jpg • Topic Dendogram.jpg • Tabel 1 Qualitative analysis of the research summary of each abstract The data are available under the terms of the Creative Commons Attribution 4.0 International license (CC-BY 4.0).
