# Supplementary material for: Ginkgo Biloba as a niche theme cognitive enhancer agent, 1420 dokumen of Scopus database. A bibliometric study from 1988 to 2024
Source: F1000Res. 2025 Oct 15;14:275. Originally published 2025 Mar 7. [Version 3] doi: 10.12688/f1000research.160416.3 (PMC12569511; doi:10.12688/f1000research.160416.3)
Supplement: Peer review history

# Reviewer report: Reviewer response for version 3

**DOI:** 10.5256/f1000research.188435.r424445

**Published:** 2025-10-28

**Authors:** Arne Reimers

**Affiliations:** 1 Department of Clinical Chemistry and Pharmacology, Division of Laboratory Medicine, Lund University, Lund, Skåne County, Sweden

The authors have resolved most of the issues I outlined in my previous review, but not all. For example, the part about fig. 13 (p12, staring with 'According to Figure 13, on the thematic map based on the title...') which the authors state has been revised, contains the exact same wording as the previous version. In other words, it has not been revised. Also, the discussion still focuses too much on Ginko biloba and too little on the authors' own findings. While these and a few other weaknesses reduce the overall quality of the paper, it appears acceptable for indexing.

Is the work clearly and accurately presented and does it cite the current literature?

Partly

If applicable, is the statistical analysis and its interpretation appropriate?

Not applicable

Are all the source data underlying the results available to ensure full reproducibility?

Yes

Is the study design appropriate and is the work technically sound?

Yes

Are the conclusions drawn adequately supported by the results?

Partly

Are sufficient details of methods and analysis provided to allow replication by others?

Partly

Reviewer Expertise:

Clinical neuro- and psychopharmacology

I confirm that I have read this submission and believe that I have an appropriate level of expertise to confirm that it is of an acceptable scientific standard.

# Reviewer report: Reviewer response for version 2

**DOI:** 10.5256/f1000research.181956.r397709

**Published:** 2025-08-22

**Authors:** Arne Reimers

**Affiliations:** 1 Department of Clinical Chemistry and Pharmacology, Division of Laboratory Medicine, Lund University, Lund, Skåne County, Sweden

This bibliometric study aims at analyzing trends and the current status of published research on Ginkgo biloba. It uses different outcome variables and visualization methods.

 There are countless small issues that make this paper difficult to read and understand. For example:

 Abstract, results and discussion: this part is supposed to reflect the study's findings. However, the authors state some arbitrarily chosen historical facts about the medical use of Ginkgo biloba.

 Abstract, conclusion: instead of a conclusion, the authors state the aims of the study.

 The title of this paper says 1420 documents. Also, the list of references consists of 1420 documents. However, in the text it is stated that 1414 documents were retrieved. This should be resolved.

 P4: the authors state that they retrieved 1414 relevant documents. Please state how 'relevant' was defined and how many irrelevant documents were excluded.

 P4: according to the authors, figure 1 shows that the annual numbers of papers culminated in 61 papers by 2023. From what I can see, the annual number culminated with 130 papers in 2014.

 P4: The authors state that the journal Psychopharmacology accepts many manuscripts. It is unclear what is meant by 'many'. Does it have a high acceptance rate? Does it publish a high number of papers per year? (if so, please quantify). Or does it mean that the journal accepts a broad variety of subjects or article types?

 P5: 'Ranking three is the Journal of Behavioral Brain Research. This contains information regarding the Journal of Behavioral Brain Research.' This does not make sense, please rephrase.

 P5: 'the journal titled Identification of separable cognitive factors in schizophrenia, authored by Keith H. Nuechterlein (…). The journal's abstract states that (…)'. I believe that the word 'journal' should be changed to 'article'.

 P6: 'two major receptors that function as primary excitatory neurotransmitters'. Receptors do not function as neurotransmitters. Please rephrase.

 Figure 4: This figure is difficult to understand without an explanation. Please explain in the text what dim1 and dim2 are. Further, clusters 1 and 2 appear to be only one single point each, so why are they called clusters? On the other hand, the articles displayed in the figure form a cluster, but this is not mentioned in the text. Moreover, the text mentions a study by Difede et al. (2013). In the figure, there is no study by Difede published in 2013.

 P8: 'shows the percentage of documents classified into different subject areas'. Please state how that classification was done.

 P9: 'indicates that the examined areas remained unassociated with the other regions delineated by the edges'. That does not make sense. Please rephrase.

 P9/10: 'according to Figure 11, in the overlay visualization, it appears that the keywords that are being researched a lot approaching 2016 are the parts colored yellow, namely gluthahione, malonaldehyde, metabolism, prescription, major clinical study, randomized controlled trial (t, addiction, student, students, universities, oxidative stress, chemistry, and nerve degeneration.' To me, it appears that the most researched key words are the green ones in the middle.

 P12: 'According to Figure 13, on the thematic map based on the title, the following is an explanation for each keyword in each quadrant in the thematic map resulting from the bibliometric analysis. Here is an explanation of the meaning of each quadrant in the thematic map, and examples of document titles relevant to keywords in each quadrant.' This does not make sense, please rephrase. 

 Figure 13 looks like a screen shot and should be replaced or at least be cropped to the figure itself.

 Results and discussion: the first two sentences are already stated in the introduction and should be deleted. The discussion does not refer to the authors' (= this study's) own findings but represents a medical/historical review of Ginkgo biloba. It needs to be re-written with a clear focus on the bibliometric findings.

 Points that must be addressed to make the article scientifically sound:

 The abstract and the discussion need to be re-written. The manuscript body needs thorough revision. As a minimum, all issues mentioned above should be resolved.

 Note: Almost all figures are impossible to read due to very low resolution. I do not know whether this is the publisher's fault. Fortunately, the authors provide a link to a repository where the figures are available in high resolution.

Is the work clearly and accurately presented and does it cite the current literature?

Partly

If applicable, is the statistical analysis and its interpretation appropriate?

Not applicable

Are all the source data underlying the results available to ensure full reproducibility?

Yes

Is the study design appropriate and is the work technically sound?

Yes

Are the conclusions drawn adequately supported by the results?

Partly

Are sufficient details of methods and analysis provided to allow replication by others?

Partly

Reviewer Expertise:

Clinical neuro- and psychopharmacology

I confirm that I have read this submission and believe that I have an appropriate level of expertise to confirm that it is of an acceptable scientific standard, however I have significant reservations, as outlined above.

# Response

**Published:** 2025-09-18

**Authors:** ARMAN YURISALDI SALEH

**Affiliations:** Neurology, Universitas Pembangunan Nasional Veteran Jakarta, Jakarta, Special Capital Region of Jakarta, Indonesia

Thank you to the reviewers for their kindness in providing feedback and suggestions.

 This bibliometric study aims at analyzing trends and the current status of published research on Ginkgo biloba. It uses different outcome variables and visualization methods.

 There are countless small issues that make this paper difficult to read and understand. For example:

 Abstract, results and discussion: this part is supposed to reflect the study's findings. However, the authors state some arbitrarily chosen historical facts about the medical use of Ginkgo biloba.

 Abstract, conclusion: instead of a conclusion, the authors state the aims of the study.

***Thank you for your very constructive feedback. We have revised the abstract, results, and discussion sections to focus more on the study's main findings and removed irrelevant historical information. Furthermore, the conclusion section of the abstract has been revised to reflect the study's results and implications, rather than simply the research objectives.***

 The title of this paper says 1420 documents. Also, the list of references consists of 1420 documents. However, in the text it is stated that 1414 documents were retrieved. This should be resolved.

***We have revised it according to the reviewer's suggestions.***

 P4: the authors state that they retrieved 1414 relevant documents. Please state how 'relevant' was defined and how many irrelevant documents were excluded.

***A search query was executed using “cognitive enhancer” in the TITLE-ABS-KEY field. This resulted in the retrieval of 1420 relevant documents. These documents were exported and stored in .csv format for further bibliometric analysis. The exported data was then processed using Biblioshiny and VOSviewer, which enabled visualization of research trends, co-authorship networks, and keyword clustering.***

 P4: according to the authors, figure 1 shows that the annual numbers of papers culminated in 61 papers by 2023. From what I can see, the annual number culminated with 130 papers in 2014.

***We initially wanted to display data based on the most recent year, but based on reviewer feedback, we have revised this to 130 papers in 2014.***

 P4: The authors state that the journal Psychopharmacology accepts many manuscripts. It is unclear what is meant by 'many'. Does it have a high acceptance rate? Does it publish a high number of papers per year? (if so, please quantify). Or does it mean that the journal accepts a broad variety of subjects or article types?

***We have revised it according to the reviewer's suggestions.***

 P5: 'Ranking three is the Journal of Behavioral Brain Research. This contains information regarding the Journal of Behavioral Brain Research.' This does not make sense, please rephrase.

 We have revised it according to the reviewer's suggestions.

 P5: 'the journal titled Identification of separable cognitive factors in schizophrenia, authored by Keith H. Nuechterlein (…). The journal's abstract states that (…)'. I believe that the word 'journal' should be changed to 'article'.

***We have revised it according to the reviewer's suggestions.***

 P6: 'two major receptors that function as primary excitatory neurotransmitters'. Receptors do not function as neurotransmitters. Please rephrase.

***We have revised it according to the reviewer's suggestions.***

 Figure 4: This figure is difficult to understand without an explanation. Please explain in the text what dim1 and dim2 are. Further, clusters 1 and 2 appear to be only one single point each, so why are they called clusters? On the other hand, the articles displayed in the figure form a cluster, but this is not mentioned in the text. Moreover, the text mentions a study by Difede et al. (2013). In the figure, there is no study by Difede published in 2013.

***We have revised it according to the reviewer's suggestions.***

 P8: 'shows the percentage of documents classified into different subject areas'. Please state how that classification was done.

***Documents were classified into subject areas based on the primary indexing category assigned by Scopus. This classification utilized publication metadata—such as journal title, keywords, and abstract content—to determine the dominant field of each document. For visualization purposes, each document was counted once under its main subject area to avoid duplication across categories.***

 P9: 'indicates that the examined areas remained unassociated with the other regions delineated by the edges'. That does not make sense. Please rephrase.

 We have revised it according to the reviewer's suggestions.

 P9/10: 'according to Figure 11, in the overlay visualization, it appears that the keywords that are being researched a lot approaching 2016 are the parts colored yellow, namely gluthahione, malonaldehyde, metabolism, prescription, major clinical study, randomized controlled trial (t, addiction, student, students, universities, oxidative stress, chemistry, and nerve degeneration.' To me, it appears that the most researched key words are the green ones in the middle.

***we took the keywords from the latest results, namely in 2016, the yellow color is in the picture***

 P12: 'According to Figure 13, on the thematic map based on the title, the following is an explanation for each keyword in each quadrant in the thematic map resulting from the bibliometric analysis. Here is an explanation of the meaning of each quadrant in the thematic map, and examples of document titles relevant to keywords in each quadrant.' This does not make sense, please rephrase. 

***We have revised it according to the reviewer's suggestions.***

 Figure 13 looks like a screen shot and should be replaced or at least be cropped to the figure itself.

***All data we used in the study have been uploaded to The article references an extended dataset on Figshare : https://doi.org/10.6084/m9.figshare.28081709.v1 and can be accessed publicly***

 Results and discussion: the first two sentences are already stated in the introduction and should be deleted. The discussion does not refer to the authors' (= this study's) own findings but represents a medical/historical review of Ginkgo biloba. It needs to be re-written with a clear focus on the bibliometric findings.

***We have revised it according to the reviewer's suggestions.***

 Points that must be addressed to make the article scientifically sound:

 The abstract and the discussion need to be re-written. The manuscript body needs thorough revision. As a minimum, all issues mentioned above should be resolved.

 We have revised the manuscript according to the reviewer's request and have also submitted the new version of the manuscript.

 Note: Almost all figures are impossible to read due to very low resolution. I do not know whether this is the publisher's fault. Fortunately, the authors provide a link to a repository where the figures are available in high resolution.

***All data we used in the study have been uploaded to The article references an extended dataset on Figshare : https://doi.org/10.6084/m9.figshare.28081709.v1 and can be accessed publicly***

# Reviewer report: Reviewer response for version 2

**DOI:** 10.5256/f1000research.181956.r385013

**Published:** 2025-08-09

**Authors:** Tasawar Iqbal

**Affiliations:** 1 University of Agriculture Faisalabad, Faisalabad, Punjab, Pakistan

Accept the article.

Is the work clearly and accurately presented and does it cite the current literature?

Partly

If applicable, is the statistical analysis and its interpretation appropriate?

Partly

Are all the source data underlying the results available to ensure full reproducibility?

Partly

Is the study design appropriate and is the work technically sound?

Partly

Are the conclusions drawn adequately supported by the results?

Partly

Are sufficient details of methods and analysis provided to allow replication by others?

Partly

Reviewer Expertise:

Pharmacology, Phytochemical, Rheumatoid Arthritis, Plants base Medicine, Animal related study, Different Disease treatment, Drug Delivery, plant study, insect physiology, Mushroom related study, Entomology, Poultry Management, Zoology, Microbiology.

I confirm that I have read this submission and believe that I have an appropriate level of expertise to confirm that it is of an acceptable scientific standard.

# Reviewer report: Reviewer response for version 1

**DOI:** 10.5256/f1000research.176313.r380845

**Published:** 2025-05-10

**Authors:** Tasawar Iqbal

**Affiliations:** 1 University of Agriculture Faisalabad, Faisalabad, Punjab, Pakistan

**Summary of the Article**

 This manuscript presents a bibliometric analysis of literature published from 1988 to 2024 focusing on *Ginkgo biloba* as a cognitive enhancer. The study uses data sourced from the Scopus database and analyzes trends, influential authors, key journals, and subject areas using Biblioshiny and VOSviewer tools. The authors identify niche, motor, and emerging themes using thematic mapping and network visualization to assess the evolution of research topics over time. Qualitative discussions are included on the traditional and pharmacological uses of *Ginkgo biloba*, with citations of clinical trial data and historical usage.

**Evaluation of Core Criteria**

**1. Is the work clearly and accurately presented and does it cite the current literature?**

**Answer: Partly**

**Comments:**

- The manuscript is generally well-structured and references a wide range of studies, including both foundational and recent works. However, some citations appear incorrectly formatted or poorly integrated (e.g., inconsistent referencing of citation numbers, limited context around cited findings).
- The discussion on Ginkgo biloba's pharmacological mechanisms includes up-to-date research but occasionally blends bibliometric content with pharmacological summaries without clear separation.
  Recommendation: Improve citation clarity and maintain a stricter distinction between bibliometric analysis and pharmacological narrative.

**2. Is the study design appropriate and is the work technically sound?**

**Answer: Partly**

**Comments:**

- The use of bibliometric tools (Scopus, Biblioshiny, VOSviewer) is methodologically appropriate.
- However, the design lacks details on inclusion/exclusion criteria, filtering strategies, and search limitations (e.g., language or document types), which are critical to ensure replicability and to reduce selection bias.
- Furthermore, the decision to not apply any filters (as stated) weakens the rigor of the bibliometric scope and could introduce irrelevant data noise.
  Recommendation: The authors should specify search criteria more clearly and provide justification for their decision to include unfiltered results.

**3. Are sufficient details of methods and analysis provided to allow replication by others?**

**Answer: Partly**

**Comments:**

- While the tools used are named, the description of the search strategy is too vague.
- The article mentions keyword use (e.g., “cognitive enhancer”) but does not provide exact queries or document identifiers for replication.
- The structure and format of exported data files are listed, but there is no access to the raw search results or scripts used for Biblioshiny or VOSviewer analysis.
  Recommendation: Include complete search strings, filters (if any), time range parameters, and preferably a supplementary dataset or repository link to raw export files.

**4. If applicable, is the statistical analysis and its interpretation appropriate?**

**Answer: Partly**

**Comments:**

- The article primarily uses descriptive statistics and visualization tools, which are valid for bibliometric analysis.
- However, no inferential statistics or statistical validation of clusters/themes is presented, nor are confidence measures or citation impact normalization addressed.
  Recommendation: Include basic bibliometric performance metrics (e.g., h-index, average citations per item) and clarify the method used to define cluster themes (e.g., co-occurrence thresholds).

**5. Are all the source data underlying the results available to ensure full reproducibility?**

**Answer: Partly**

**Comments:**

- The article references an extended dataset on Figshare https://doi.org/10.6084/m9.figshare.28081709.v1, which contains visualizations and thematic maps.
- However, the raw Scopus CSV or RIS files used for analysis are not provided, and key elements such as search queries or metadata fields are not included.
  Recommendation: The authors should upload all exported files and search history from Scopus as supplementary material or Figshare content.

**6. Are the conclusions drawn adequately supported by the results?**

**Answer: Partly**

**Comments:**

- The conclusions reflect findings from both the bibliometric mapping and the qualitative narrative on Ginkgo biloba's therapeutic effects.
- However, some extrapolations such as effectiveness and safety in clinical contexts—are based on limited bibliometric evidence, rather than systematic reviews or meta-analyses.
  Recommendation: Limit claims on clinical efficacy unless substantiated by the specific bibliometric content reviewed; distinguish bibliometric findings from clinical interpretation.

**Overall Strengths**

- Addresses a valuable niche topic within cognitive enhancement research.
- Employs credible bibliometric tools and platforms (Scopus, Biblioshiny, VOSviewer).
- Provides useful visualization and clustering analysis.

**Major Revisions Required to be Scientifically Sound**

1. Clarify and document the search methodology in full detail to ensure reproducibility.
2. Provide access to the raw Scopus data exports and search strings.
3. Justify the absence of filters or explain how the lack of filtering does not compromise data validity.
4. Avoid merging clinical conclusions with bibliometric findings unless systematically substantiated.
5. Improve citation consistency and integrate references more clearly throughout the discussion.

Is the work clearly and accurately presented and does it cite the current literature?

Partly

If applicable, is the statistical analysis and its interpretation appropriate?

Partly

Are all the source data underlying the results available to ensure full reproducibility?

Partly

Is the study design appropriate and is the work technically sound?

Partly

Are the conclusions drawn adequately supported by the results?

Partly

Are sufficient details of methods and analysis provided to allow replication by others?

Partly

Reviewer Expertise:

Pharmacology, Phytochemical, Rheumatoid Arthritis, Plants base Medicine, Animal related study, Different Disease treatment, Drug Delivery, plant study, insect physiology, Mushroom related study, Entomology, Poultry Management, Zoology, Microbiology.

I confirm that I have read this submission and believe that I have an appropriate level of expertise to confirm that it is of an acceptable scientific standard, however I have significant reservations, as outlined above.

# Response

**Published:** 2025-05-12

**Authors:** ARMAN YURISALDI SALEH

**Affiliations:** Neurology, Universitas Pembangunan Nasional Veteran Jakarta, Jakarta, Special Capital Region of Jakarta, Indonesia

Thank you for the detailed and constructive feedback during the peer review process. 

 Major Revisions Required to be Scientifically Sound

 1. Clarify and document the search methodology in full detail to ensure reproducibility.

 2. Provide access to the raw Scopus data exports and search strings.

 Justify the absence of filters or explain how the lack of filtering does not compromise data

 validity.

 3.

 Avoid merging clinical conclusions with bibliometric findings unless systematically

 substantiated.

 4.

 Improve citation consistency and integrate references more clearly throughout the

 discussion.

 Peer Review :

 1. We have revised the manuscript according to the reviewer's request and have also submitted the new version of the manuscript.

 2. All data we used in the study have been uploaded to The article references an extended dataset on Figshare : https://doi.org/10.6084/m9.figshare.28081709.v1 and can be accessed publicly

 3. We have revised the manuscript according to the reviewer's request and have also submitted the new version of the manuscript.

 4. All citations are in Table 1 Qualitative analysis of the research summary of each abstract that has been uploaded is also in Figshare : https://doi.org/10.6084/m9.figshare.28081709.v1 and can be accessed publicly

 Thank you again for the opportunity to enhance the quality of this manuscript.
